# Production and use of rapid responses during the COVID-19 pandemic in Quebec (Canada): perspectives from evidence synthesis producers and decision makers

**DOI:** 10.1186/s12961-024-01105-x

**Published:** 2024-02-13

**Authors:** Esther McSween-Cadieux, Julie Lane, Quan Nha Hong, Andrée-Anne Houle, François Lauzier-Jobin, Eliane Saint-Pierre Mousset, Ollivier Prigent, Saliha Ziam, Thomas Poder, Alain Lesage, Pierre Dagenais

**Affiliations:** 1 Department of School and Social Adaptation Studies, Faculty of Education, Université de Sherbrooke, Sherbrooke, Canada; 2 Centre RBC d’expertise Universitaire en Santé Mentale, Université de Sherbrooke, Sherbrooke, Canada; 3 School of Rehabilitation, Université de Montréal, Montreal, Canada; 4 Department of Psychoeducation, Université de Sherbrooke, Sherbrooke, Canada; 5 School of Business Administration, Université TÉLUQ, Montreal, Canada; 6 Centre de Recherche de l’Institut Universitaire en Santé Mentale de Montréal (CR-IUSMM), CIUSSS-de-l’Est-de-l’île-de-Montréal, Montreal, Canada; 7 Department of Management, Evaluation and Health Policy, School of Public Health, University of Montreal, Montreal, Canada; 8 Department of Medicine, Faculty of Medicine and Health Science, University of Sherbrooke, Sherbrooke, Canada

**Keywords:** Rapid responses, Rapid evidence synthesis, Evidence-informed decision-making, Evidence use, COVID-19, Pandemic, Quebec, Canada

## Abstract

**Background:**

The COVID-19 pandemic has required evidence to be made available more rapidly than usual, in order to meet the needs of decision makers in a timely manner. These exceptional circumstances have caused significant challenges for organizations and teams responsible for evidence synthesis. They had to adapt to provide rapid responses to support decision-making. This study aimed to document (1) the challenges and adaptations made to produce rapid responses during the pandemic, (2) their perceived usefulness, reported use and factors influencing their use and (3) the methodological adaptations made to produce rapid responses.

**Methods:**

A qualitative study was conducted in 2021 with eight organizations in the health and social services system in Quebec (Canada), including three institutes with a provincial mandate. Data collection included focus groups (*n* = 9 groups in 8 organizations with 64 participants), interviews with decision makers (*n *= 12), and a document analysis of COVID-19 rapid responses (*n* = 128). A thematic analysis of qualitative data (objectives 1 and 2) and a descriptive analysis of documents (objective 3) were conducted.

**Results:**

The results highlight the teams and organizations’ agility to deal with the many challenges encountered during the pandemic (e.g., increased their workloads, adoption of new technological tools or work processes, improved collaboration, development of scientific monitoring, adaptation of evidence synthesis methodologies and products). The challenge of balancing rigor and speed was reported by teams and organizations. When available at the right time, rapid responses have been reported as a useful tool for informing or justifying decisions in a context of uncertainty. Several factors that may influence their use were identified (e.g., clearly identify needs, interactions with producers, perceived rigor and credibility, precise and feasible recommendations). Certain trends in the methodological approaches used to speed up the evidence synthesis process were identified.

**Conclusions:**

This study documented rapid responses producers’ experiences during the COVID-19 pandemic in Quebec, and decision makers who requested, consulted, or used these products. Potential areas of improvements are identified such as reinforce coordination, improve communication loops, clarify guidelines or methodological benchmarks, and enhance utility of rapid response products for decision makers.

## Background

It is important for health and social services decision makers to have access to quality and timely evidence to inform decision-making, particularly during health emergencies [1, 2]. The COVID-19 pandemic has posed significant challenges to evidence-informed decision-making and the development of evidence syntheses to support it [3]. Because of the rapid spread of the virus, the burden of morbidity and mortality, and the significant implications for health systems resources [4], important decisions had to be made urgently. This situation has created a strong incentive for policy-makers to use evidence [3] to reduce the risk of transmission and limit mortality.

### Evidence-informed decision-making during COVID-19

The knowledge base of this new coronavirus was poor at the outset of the pandemic due to its specific features, which made comparisons with previous outbreaks difficult [5]. More importantly, evidence was also constantly evolving as researchers gained a better understanding of the virus. Thus, an increasing number of studies on COVID-19 were weekly available, with many not peer-reviewed yet. In other words, critical decisions had to be made urgently based on evidence that were sparse, inconclusive, uncertain, of variable quality, and evolving rapidly [6–8]. The challenge of developing evidence-based recommendations was therefore significant during the early months of the pandemic [6]. This put a lot of pressure on the teams responsible for producing evidence syntheses and making recommendations to decision-makers, health professionals and the public. They had to provide timely guidelines in a context of great uncertainty, in an emerging field, and using sometimes unconventional data [3, 4].

### Increasing demands for rapid responses

During the pandemic, decision makers had evidence needs that could not be met by traditional synthesis methods such as systematic reviews. Indeed, the time, production cost, and expertise required to conduct these types of evidence syntheses are significant barriers when decisions must be made quickly [9–12]. The various teams or units responsible for knowledge synthesis and clinical guideline development within health systems organizations had to adapt their working methods [8, 13]. Their evidence synthesis methods had to be both fast and responsive while being rigorous because of the implications that their knowledge products could have. These had to be done faster than ever, in a few weeks, a few days or sometimes in a few hours [6, 14]. These units needed to build skilled and motivated multidisciplinary teams, while ensuring transparency and appropriate management of conflicts of interest [15].

Also, several countries have had to adapt their infrastructure services for evidence synthesis. Several initiatives have been developed such as rapid responses units and systematic reviews, rapid reviews, primary studies, or recommendations repositories (e.g. eCOVID-19RecMap, COVID-END, WHO COVID-19, the COVID-NMA initiative, LitCovid, L·OVE platform) [14, 16–18].

### Methodological adaptations of rapid evidence synthesis

Despite the growing interest in rapid evidence synthesis products over the past decade and the methodological guidelines emergence over the past five years [2, 19–21], there is no clear consensus on the terminology, definition and methods characterizing them [10, 22–27]. The terms most frequently used to name these approaches are *rapid review, rapid responses*, *rapid evidence assessments*, *rapid systematic reviews,* and *rapid health technology* [28]. During the pandemic, Cochrane published interim recommendations offering specific guidance on steps and considerations for conducting rapid reviews, and minimum standards [21].

In this study, we use the term *rapid response* rather than *rapid review*, to recognize the range of methodological approaches used during the pandemic to produce evidence syntheses to support emergency decision-making. Thus, rapid responses (RR) refer to different knowledge synthesis products that were produced in an accelerated and abbreviated manner on different topics related with the COVID-19 pandemic. For example, RR could be on the effectiveness of drugs (e.g., remdesivir) for the treatment of COVID-19 or of protective measures (e.g., mask) to prevent the spread of COVID-19.

To speed up the evidence synthesis process, some compromises can be made in terms of the methodological standards expected during these different steps [29]. Some shortcuts are used such as narrowing the literature search or the eligibility criteria (types of specifications, year of publication, language), using only one reviewer for study selection, data extraction and quality assessment, or conducting a narrative synthesis [8, 27, 28, 30–32]. As presented in the STARR Decision Tool, methodological approaches to conducting rapid reviews can vary based on several dimensions: the interaction with commissioners, the scope and search of the evidence, the methods used for data extraction and synthesis, and the reporting of the methods [33]. In a precautionary approach, certain shortcuts are recommended to be avoided, such as not assessing risk of bias or involving only one reviewer in the selection, extraction, or risk of bias assessment [21, 32, 34].

In general, methodological adaptations reduce production time and save resources. However, they can have implications on conclusions’ validity [28, 29]. The shortcuts used in rapid methods bear the risk that results may be potentially less reliable than those of traditional systematic reviews [35, 36]. Since they are primarily carried out to inform an urgent decision that may have a significant impact on the public, it is essential to be transparent about the adaptations made and their potential impacts.

Because of their role in decision-making, further study of methodological adaptations to produce RRs, their characteristics and use, especially in the context of a public health crisis, are needed [37, 38]. Since this will probably not be our last public health crisis, we must learn from it and share the lessons learned about rapid evidence synthesis [6].

## Objectives

This study aimed to document both the perspectives of professionals from health systems teams and organizations in Quebec, including institutes with provincial mission, and those of decision-makers on RRs produced during the COVID-19 pandemic. Quebec is the second largest province in Canada and has a special status in Canada as a French-speaking province. In Canada, the governance of health and public health is decentralized. The provinces have primary jurisdiction over health care. Quebec. It also has its own health technology assessment (HTA) agency and teams producing rapid responses. Quebec was the province that was the most impacted by the first wave of the pandemic [39].

This research project had three specific objectives:

- **Objective 1.** Document the challenges and adaptations made by evidence synthesis producers to produce rapid responses during the pandemic in Quebec.
- **Objective 2.** Document the perceived usefulness, types of use and factors influencing the use of rapid responses by decision-makers in Quebec.
- **Objective 3.** Document the methodological adaptations of rapid responses made during the pandemic by health and social services organizations in Quebec.

## Methods

### Study design and setting

This study was developed in response to needs identified by the project partners and was conducted in close collaboration with them. Participating organizations include two national institutes (supporting the Ministry of Health and Social Services (MHSS)), regional public health authorities, and regional health and social services institutions), one province-wide research institute, and five HTA units involved in health and social services. In Quebec, these units operate in integrated university health and social services centers (IUHSSC/IHSSC) to support decision-making through evidence synthesis [40]. The Quebec health system comprises twenty-two integrated regional centers, with or without a university mission. These integrated centers report to the MHSS and are responsible for delivering a range of health and social services in their designated regional territory in the province.

For recruitment, an email was sent by the principal investigator (JL) to 12 organizations’ managers to invite them to collaborate in this research. Eight of them agreed to participate in the data collection and the others wanted to be collaborators in the project as knowledge users. The participating organizations were then asked to specify their expected role. A person was designated within each organization to act as a key contact and facilitate the project in their milieu (e.g., methodology feedback, recruitment and data collection coordination, report review).

### Data collection

#### Objective 1: focus groups with rapid responses’ producers

For the first objective, focus groups were conducted via videoconference with HTA teams and organizations in May and June 2021. Their duration varied between 90 to 120 min. Participants were involved in the production of a RR project (e.g., literature search, data analysis, product writing and dissemination, project coordination) during the pandemic on a topic related to COVID-19.

The questions concerned the mandate and structure of teams or organizations and the perceived impacts of the pandemic on their work processes and methods. The main challenges experienced during the pandemic, methodological adaptations in evidence synthesis and lessons learned from their experience were also explored. These focus groups were conducted by the principal investigator (JL) and the project coordinator (EMC). They were recorded and fully transcribed.

#### Objective 2: semi-structured interviews with rapid responses’ users

For the second objective, semi-structured interviews were conducted via videoconference with RRs’ users in October and November 2021. Questions concerned their appreciation and use of RRs for decision-making in an emergency context and factors that could have influenced their use and recommendations application. The interviews were conducted by a research professional (AAH) and were recorded and fully transcribed.

#### Objective 3: document analysis

For the third objective, a document analysis of RR methodological adaptations was conducted. RR products published between March 13, 2020, and April 30, 2021, were identified from documents provided by the organizations and websites search. Documents were selected based on the following criteria: (1) documents had to include a description of the methodology used in relation to the evaluation question, and (2) the methodology had to include an evidence synthesis.

The data extracted were based on the rapid review steps, mainly literature search, citation screening, data extraction, quality assessment and data synthesis [14]. The following data were extracted into a Microsoft Excel file: publication date, inter-organizational collaboration (dichotomous), requesters (nominal), product type (nominal), research questions (nominal), target audience (nominal), selection criteria (dichotomous), literature search strategy (e.g., databases used, grey literature search, restrictions used in literature search (dichotomous)), implication of a librarian (dichotomous), document selection and data extraction (e.g., number of reviewers involved (numeric), quality assessment and certainty of evidence (dichotomous), methods of synthesis (nominal), revisions and consultations (dichotomous), number of documents retained (numerical), nature of documents retained (nominal), number of references (numerical), number of pages (numerical), and methodological limitations (dichotomous) and warning (dichotomous). Four people were involved in the data extraction (QNH, EMC, OP, ESPM). A second person verified the extracted data for 20% of the documents to ensure accuracy and to clarify the information to be extracted.

### Data analysis

For the first two objectives, a thematic analysis of the qualitative data was conducted. The themes were developed inductively. A six-step iterative process and two coding cycles were followed [41]. The first step consisted of preparation and familiarization with the data. The recordings were listened and the verbatim were reviewed. Subsequently, the verbatim were added into NVivo 13©. In the second step, a preliminary list of initial codes was created from the data. Next, a first round of (structural) coding was performed and themes were identified. In the fourth step, a second round of coding (pattern) was performed in which the themes were examined by reading the excerpts from each theme and making connections between the data. Next, the analysis consisted of naming, defining, and organizing the themes in a consistent manner. The final step was to write up the results, which involved selecting excerpts to support the themes and making connections to the research questions.

For the third objective, a descriptive analysis of the data extracted from the retained RR products was performed. For numerical data (e.g., number of documents retained, number of pages), medians and ranges were calculated.

## Results

### Objective 1: Perspectives of rapid responses’ producers

Nine focus groups with 64 professionals were conducted: one group in seven organizations and two groups in one organization. The groups’ size ranged from two to thirteen people. The individuals who participated were scientific professionals or advisors, health and social services technology assessment and evaluation professionals, knowledge mobilization advisors, researchers, information specialists, HTA units’ scientific leaders and team managers.

The challenges and adaptations made by the teams in charge of producing RRs during the pandemic are presented in three categories (personal and professional, organizational, and methodological) and summarized in Table 1.

**Table 1:** Main challenges and adaptations made by evidence synthesis producers

Challenges	Adaptations
Personal and professional level	
Urgent requests for RR	• Increase workloads, work pace and worked hours
New realities of remote working	• Adoption of new work processes and tools • Availability of appropriate technology resources (e.g., collaborative tools)
Organizational level	
Duplication of efforts between organizations	• Collaborations within and between organizations • Better coordination with stakeholders
Highly mediatized context	• Improvement of clarity and precision of communication
Lack of awareness of the role of teams that producde syntheses	• Clarification of mandate and team positioning • Demonstration of their added value to the decision-making process
Methodological level	
Limited interaction with requesters	• Navigating uncertainty and moving forward despite limited information
Limited robust evidence and constantly evolving evidence	• Development of scientific monitoring mechanisms • Use of various data collection strategies (e.g., capture experiential and contextual evidence)
Balance between speed of production and methodological rigour	• Adaptation of the evidence products developed (e.g., new templates) • Methodology tailored to the nature of the request • Statement on RR limitations in the final document • Updating documents

#### Personal and professional category

Several participants noted that the knowledge needs of decision-makers were urgent and that evidence synthesis had to be produced much more quickly than usual. The pandemic exceptional context induced a strong sense of purpose among many of the participants. Several mentioned a certain satisfaction in being able to provide information that met the needs of decision-makers at a specific time. However, to adapt and meet the needs, they had to **increase workloads, work pace and work hours**, which represented challenges. The sense of urgency and desire to contribute had negative impacts on the physical and mental health of several participants. Work-family balance, especially in a context where parents were working from home and children were staying at home, was an important issue to highlight.

*"There was still a great risk to the physical and mental health of our team. We had to keep the light on, the demands were considerable and if we had listened to all the requests, we would have worked 24 hours a day [...]" (Focus group 2)*

A recommendation made by several participants was the importance of **providing support to professionals** regarding their psychological and physical health, particularly from the immediate supervisor.

The transition to telework was an important adjustment implemented in the organizations. Professionals had to quickly adapt to this new reality and **adopt new work processes and tools**. On a personal level, one negative dimension of telework was the limited informal exchanges and a certain feeling of losing contact with colleagues. Some people mentioned that this may have affected their productivity and hindering spontaneity and creativity. To ensure an optimal teleworking adaptation, **early access to and availability of appropriate technology resources**, primarily collaborative tools (e.g., Covidence©, Microsoft Teams©), were mentioned as keys to success. Conversely, for some organizations, the lack of access to suitable technological tools may have affected work efficiency (e.g., no remote access to usual tools, no collaborative platforms, technical problems). For example, some people reported that they were not prepared to deal with the implications of working remotely (e.g., workspace ergonomics, appropriate tools, training, computer issues).

#### Organizational category

The pandemic response has led to the **development of collaborations** internally, between colleagues and different teams, but also externally, between departments and between organizations. Several participants noted that the pandemic has also led to **better coordination** with stakeholders. According to them, these collaborations should be maintained to ensure maximum efficiency and quality of response to decision-makers’ requests and to avoid duplicating efforts among the various HTA units and institutes in Quebec. One team that reported that they did not collaborate with other organizations because of the specificity of their requests nevertheless raised the importance of making projects more widely known:

*"So yes, there are some challenges [to inter-institutional collaboration], but I think it would still be worthwhile for people... at least to be aware of what’s going on elsewhere and for people to be open to collaborating as well"* (Focus group 4)

The importance of effective resource management and coordination was also raised by some participants. More specifically, these concerns both the way evidence requests are managed, and the work done by professionals and organizations. It was recommended that the mandates of the various teams and organizations be clarified to avoid duplicating the work carried out, both within the organizations and between the various HTA units and institutes.

*"For me it was more the redundancy of doing the work in several directions, even in other institutions... we attended meetings, committees that were doing the same work we were doing [...]"* (Focus group 2)

Moreover, the way that requests were received or anticipated sometimes generated dissatisfaction among professionals. More specifically, some felt that they had worked in a reactive rather than proactive manner, which may have limited their responses’ effectiveness. Some professionals would have preferred to be able to anticipate decision makers’ requests rather than be in an action-reaction loop. In this regard, it is recommended to improve the mechanisms for managing the entry of requests and the mechanisms for tracking the RRs completed and thus improve accountability.

In the context of a pandemic, the reach of evidence products developed was much greater as they were more widely read. The pandemic led organizations to address urgent and uncertain topics in a highly mediatized context. This greater exposure also came from the political and private sectors for whom the evidence synthesis’ conclusions could have major implications. By having a greater media and political influence, the products brought additional pressure and responsibility on professionals and organizations. This increased focus has led some organizations to **consider the sensitive nature** of the issues more carefully and to adjust their key messages. **Improving the clarity and precision of communication** have therefore been a particularly important adaptation, prompting some to recommend that it be maintained even after the pandemic.

However, beyond the increased impact of evidence products produced, some professionals were disappointed to find that certain recommendations were not fully implemented. Many professionals were nevertheless proud of the quality and rigour of the publications produced during the crisis, among other things, because of the positive comments received from users. Some participants from few organizations, however, mentioned that they had received little feedback on their products and would be interested in learning more about their RRs’ usefulness and use.

According to several participants, the work accomplished during the pandemic has also enabled teams from different organizations to **gain recognition and to better position themselves within the decision-making process**. The pandemic also confirmed that the work done by some organizations may not be widely known. This supported the need to increase awareness of their respective mandates, expertise and the HTA units’ added value. Some considered the importance to further promote the relevance of their evidence-informed decision support services. In addition, one way to raise awareness of the evidence synthesis benefits would be to encourage a culture of evidence-informed decision-making:

*"The culture of using or relying on evidence to make these decisions needs to start at the top and be cascaded down to the managers... And right now, we don’t have that culture, so that would be the recommendation I could make... is that this evidence-based culture needs to be embedded by the leadership and pushed down as a core value." (Focus group 6)*

#### Methodological category

To expedite the evidence synthesis process, interactions with requesters appear to have been limited. Specifically, framing and clarifying the RRs mandate was often difficult:

*"We were uncomfortable, we were very aware that we were not able to meet our usual standards. It was almost impossible to go back to our requesters and try to get them to clarify their questions, because these are people who were in a crisis cell, and we didn’t have access to them." (Focus group 9)*

Despite this often restricted access to decision-makers to specify their needs, the teams had to go ahead with their RR with the information available to them.

Particularly in the early months of the pandemic, one of the major challenges participants faced was the lack of robust evidence to answer requesters’ questions. Some participants experienced frustration in getting their work done. Participants were then quickly overwhelmed by a large amount of information that was evolving very rapidly, even too quickly. New information was available continuously, making their synthesis almost obsolete by the time it was completed. This may have led to a certain discouragement among participants faced with the constantly repeating work to be done.

*"It’s always a little concerning to send a position or a response to an issue with so much uncertainty about the depth of evidence available, … knowing very well that it could change completely in a week." (Focus group 5)*

To address this, an important strategy was the **development of scientific monitoring mechanisms** to provide accelerated access to the most recent scientific publications, pre-publications and grey literature on various topics related to COVID-19. In addition, to address the challenge of evidence quality, professionals have **developed various data collection strategies**. For example, in certain organizations, some relied on experiential and contextual evidence and on stakeholders’ values and preferences. To obtain experiential evidence, they took the time to consult with clinicians and healthcare users, despite the rapidity of the expected response.

Some teams and organizations had to **adapt the type of evidence synthesis products produced** as well as the templates and had to tailor their methodology to the targeted issue and the demand type. Despite this commitment to adapt to the demands of decision makers and the context, professionals may have found it difficult to make methodological choices in the absence of a predefined approach.

*"We didn’t know what choices, what compromises to make within the methodological approach, but also adapted to the question and context of the question we had." (Focus group 9)*

In this context, the speed of product development was a major challenge. For some professionals, producing RRs conflicted with their usual way of doing things, even describing this acceleration as a change in work paradigm. Balancing speed of execution with scientific rigour was an important consideration. According to some, the methodological modifications sometimes led to a decline in methodological standards. One adaptation was to **emphasize limitations of the synthesis in the final document.** This acceleration in the speed of production was, however, recognized to be justified by the crisis context and the need for timely responses from decision makers. The latter needed to be able to orient themselves quickly, despite the imperfection of the responses produced. If information was changing rapidly, it was therefore necessary for organizations to **produce updates just as quickly**. The RRs thus became a sort of living and evolving literature:

*"...the system needs something to guide itself and if in two days, in two weeks, we have different data, we will readjust and explain it." (Focus group 7)*

### Objective 2: perspectives of rapid responses’ users

Interviews were conducted with 12 users. These are individuals who requested, personally received, or used a RR during the pandemic to inform decision-making or to respond to a knowledge need. The interviews lasted between 30 and 75 min and were conducted in October and November 2021. Four participants were from three IUHSSC/IHSSC and eight were from MHSS departmental branches or regional public health branches.

#### Usefulness of rapid responses during the pandemic

In a context where decisions had to be made before evidence could be consolidated, RRs were perceived as useful for integrating as much scientific evidence as possible into the decision-making process. According to several participants, RRs were sometimes the only product available to obtain crucial information on which to base their decisions. They were seen as essential in times of a pandemic, although they needed to be combined and integrated with other types of inputs to make the best decisions in the context. The lack of evidence robustness, due to temporary or partial data, required for decision making to combine scientific evidence with expert opinions to have the most comprehensive understanding possible. This uncertainty may have limited the influence of science at certain times, as stated by this participant:

*"In situations where there is much more established science, you rarely need to respond in a hurry and the science is much more synthesized and it plays a much more predominant role in decision-making. In a more uncertain and emerging context, it plays a smaller role, but it’s essential that it’s there." (Evidence user 12)*

Overall, the participants had a positive perception of RRs relevance, but it must be used in a well-defined context. First, they must be used to meet a specific need in a crisis where traditional evidence synthesis methodologies would not be able to provide a timely response. Second, RRs must be used within a limited scientific context that is constantly evolving and where international knowledge is in motion. Third, they should be used to address a very specific topic or decision-making need that is not necessarily generalizable to different contexts. Finally, given the specific contribution of RRs and their less comprehensive nature than traditional systematic reviews or HTA, some participants noted that it is important for decision makers to be aware of their narrow scope.

Familiarity with RRs and experience in requesting them varied among participants. For many, frequent demand of evidence synthesis product was already part of their practice, as they were accustomed to relying on scientific evidence during the decision-making process. For some, requesting RRs was not part of their regular practice before the pandemic, but the context has led to an occasional use of these reports. Some even expressed their intention to use it more often to integrate evidence into the decision-making process. Related to this, one participant noted that an underlying issue with the variable use of RRs during the pandemic is linked to the culture of evidence-based decision-making:

*"[The challenge] was much deeper and much more related to governance in public health, how science has a place or scientific advice has an explicit place or not to guide decision-making. [...] In what I perceived, it was not at all a systematic use [of science] or even a systematic reliance on research institutes, which to me is very, very concerning." (Evidence user 10)*

#### Types of use of rapid responses in decision-making

Participants appreciated the various RRs used or requested during the pandemic. The majority were very satisfied with the products. Satisfaction was mainly diminished by responses that did not come at the right time to support an urgent decision or that partially met regional needs. Participants from the regional directorates raised these issues:

*"[the RRs] could be of excellent quality, but they came much too late. [...] Otherwise, it rarely answered questions that were asked at the regional level, which is still the place where the control of transmission work was done [...]" (Evidence user 10)*

According to some participant, the use of RRs could have been limited, in part due to the lack of capacity to respond quickly to the evidence needs of decision makers:

*"[...] to me it should just be better positioned and better communicated. If people could know that they have access to a service like that... but the problem is that I think there’s also a lack of capacity, I don’t know the capacity of the [department], but it’s like the whole health system, I think there’s a lack of capacity as well to provide these responses, it’s a huge undertaking." (Evidence user 2)*

The RRs’ recommendations were generally followed, as reported by participants. However, their implementation would have been influenced by the instance with decision-making authority:

*"Between what we recommend [...] and what recommendation is put in place, it’s always going to depend on who has the final decision-making authority. [...] When the rapid response is on a public health issue, but we are not the ones who have the final decision, and this has happened. [...], Well... the recommendation is not necessarily fully implemented [...]. Most of the time, though, I would say it’s mostly implemented." (Evidence user 6)*

Because RRs were an important tool for pandemic management, participants reported using them in different ways. The main utilization reported was that RRs were helpful in thinking through decisions, though not directly leading to change. They therefore identify these products as a tool to support reflection through a multidimensional decision-making process. They have contributed to decision makers’ processes by allowing them to question their ways of doing things or to be informed of the orientations and possible options:

*"It’s always been an important input, but rarely has it been a ready-made recipe, that you just say... you take it and you apply it as is. So, it’s as if it’s always been an input in a process that required negotiation, considering dimensions that were not always scientific [...]" (Evidence user 12)*

The second most frequent reported utilization is a more strategic use of RRs to validate, reinforce or reassess actions already taken:

*"[...] It confirms what we are already doing, and we are happy with it, and it reaffirms that it is effective, efficient, and useful, or it allows us to reorient ourselves a little bit, to ask questions about the method that is proposed. (Evidence user 9)*

Also, relying on RRs would have given some weight to the decision made, when based on the formulated conclusions or recommendations, and helping to advocate ideas:

*"[...] Sometimes it helped us to defend an idea that we already had with political actors, who for other reasons, were against something, wanted to lighten the measures too much, and it helped us to support our positions." (Evidence user 12)*

According to participants, the RRs also led to more direct evidence use by informing guidelines’ development. For example, RRs have supported the development of guidelines in facilities and implementation of health measures, enabled the development of initiatives or new programs for beneficiaries or supported the purchase of devices:

*"Well from my side, it was a very positive influence anyway, it allowed us to move forward, we made some acquisition of equipment, based on that rapid response." (Evidence user 5)*

Finally, some participants identified clinical practice changes influenced by RRs, which would have produced benefits for health professionals and patients. For example, decisions informed by RRs would have led to improved protection and limited spread of the virus, changes in the safety measures, improvements in services organization, and the development of new intervention modalities (e.g., remote intervention) and other innovative solutions.

#### Factors that influence the use of rapid responses

Several factors were identified by decision makers as influencing the use of rapid response (Table 2). For example, these factors include decision-makers' openness to mobilize scientific evidence, clear identified needs, interactions with producers to ensure the RR are aligned with decision-making needs, perceived credibility and rigor of the rapid evidence synthesis process, precise and feasible recommendations, and effective dissemination strategies.

**Table 2:** Factors influencing the use of rapid responses during the pandemic

Category	Factors reported
Governance and decision-making processes	Collaboration and coordination between organizations (regional and provincial levels) to provide timely access to evidence and expertise
	Strategic positioning of evidence synthesis producers in the decision-making system (e.g., existing connections with decision-makers)
	Openness and familiarity of decision makers to rely on scientific evidence to inform decision-making processes
	Level of complexity for integrating scientific evidence into decision-making due to other considerations
Framing the demand and need for rapid response	Importance of clearly identifying the needs of decision makers and clarify the request to obtain a RR that will be useful
	Frequent interactions between requesters and producers to ensure that the RRs remain aligned with the needs of decision makers
Rapid response production process	Evidence synthesis processes’ rigor, quality, transparency and speed
	Consideration and integration of multiple evidence sources
	Credibility and trust granted by the decision makers to the team or organization producing the RR
Recommendations contained in the rapid response	Recommendations that are free of political pressure (e.g., adopting an informed and scientific posture)
	Recommendations that are not based on the opinions of individual researchers, but on a group of experts who are independent
	Feasibility of recommendations based on resources and capacity required to implement them
	Recommendations that are specific, operational, and easily identifiable by the decision maker in the document
Rapid response dissemination and knowledge translation	Efficient dissemination channels put in place to ensure that RR reach the right people quickly
	Effective visual presentation to make RR products visually appealing and easily understandable
	Disseminate evidence as soon as possible, including direct exchanges with decision makers to reduce delays while waiting for the final document

### Objective 3: methodological characteristics of rapid responses

**Fig. 1:**
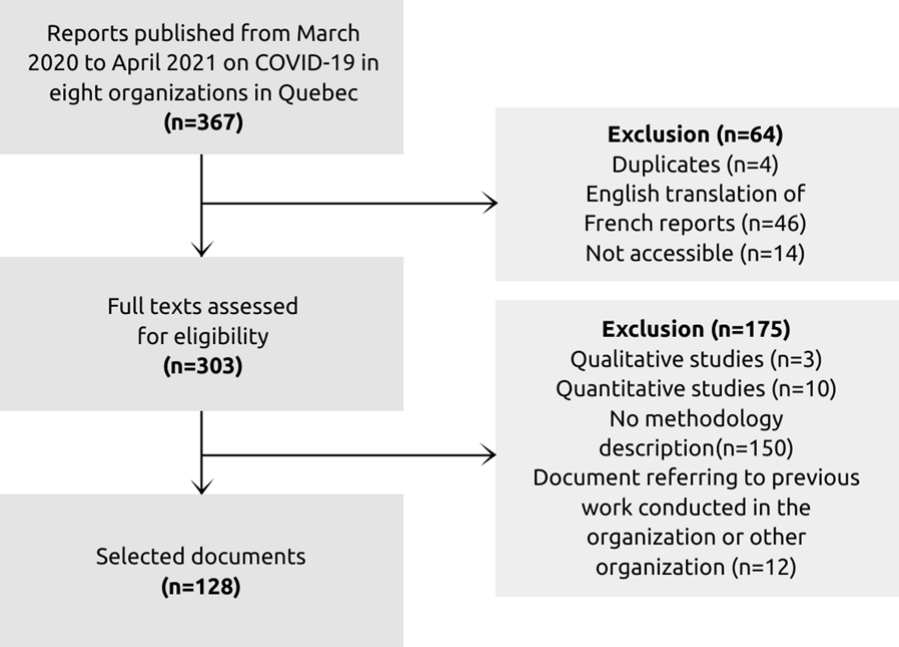
Flow diagram of the documents selected for the document analysis

The search identified 367 documents. Of these, 128 were retained for data analysis. Figure 1 shows the flow diagram, including the reasons for the exclusion of documents.

#### Characteristics of the selected documents

**Fig. 2:**
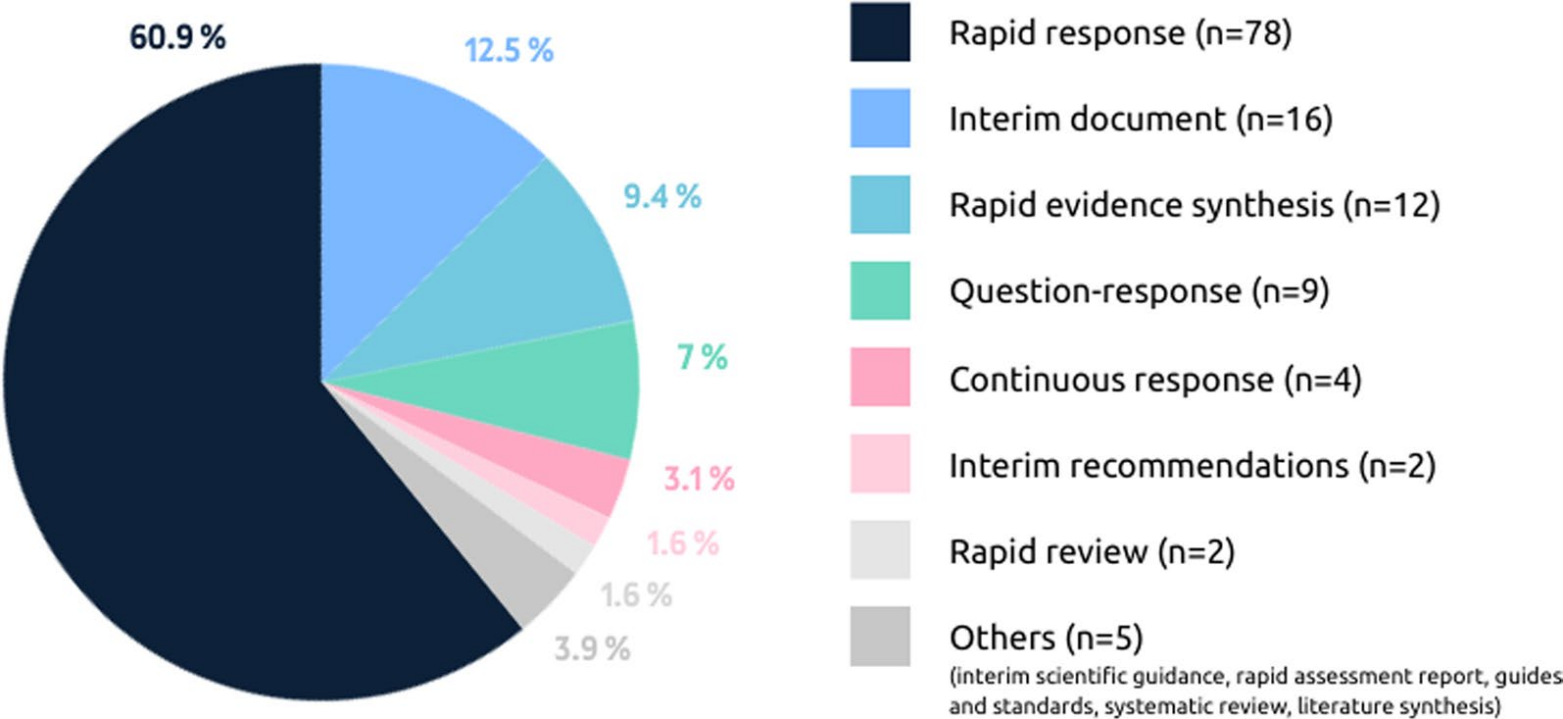
Terms used to name the rapid evidence products analyzed

Different terms were used by organizations to identify their documents (Fig. 2). The most common term was *rapid response* used in 78 documents (60.9%).

Of the 128 documents analyzed, eight (6.3%) were produced jointly by two organizations. The requester was specified in 89 reports (69.5%). The requests came from the MHSS (*n* = 71), an IUHSSC/IHSSC directorate (*n* = 11), the Public Health Direction (*n* = 3), a professional order (*n* = 1), and health professionals (*n* = 1). Two reports noted that this was an organizational initiative. The target audiences for the reports were health and social service professionals, managers, public policymakers, workers, and the general population.

Topics covered included healthcare and social services (*n* = 47), prevention and organizational measures (*n* = 24), medications (*n* = 23), impacts of COVID-19 (e.g., suicidal behaviours, food insecurity) (*n* = 12), transmission and screening (*n* = 13), and symptoms and syndromes (*n* = 9).

The length of the documents varied widely. The number of pages ranged from 2 to 129, with a median of 21.5 pages. Also, the number of references ranged from 1 to 128, with a median of 31 references. Almost all (*n* = 121; 94.5%) had a disclaimer at the beginning of the document to inform readers of the rapid evidence synthesis process, reservations about the conclusions reached, and potential future updates. However, few (*n* = 22; 17.2%) discussed the limitations of the methodology.

#### Methodological adaptations of the analyzed documents

The methodological description was succinct for most reports, ranging from one paragraph to a few pages. The analysis identified the main methodological characteristics according to the evidence synthesis stages (i.e., identification, selection, extraction, evaluation, and synthesis of data) (Table 3).

**Table 3:** Methodological characteristics reported in rapid evidence synthesis analyzed (*n* = 128)

Steps	Methodological approaches	*n* (%)
Literature search	Electronic databases searched	127 (99.2)
	Grey literature searched	126 (98.4)
	Information specialist involved	70 (54.6)
	Limit of publication language	80 (62.5)
	Limit of publication date	42 (32.8)
Citation screening	Details on number of reviewers involved in screening: - Screening done by one reviewer - Screening done by more than one reviewer (sharing, validation, or independent screening)	49 (38.3) 23 (18) 26 (20.3)
	Details on eligibility criteria	56 (43.8)
	Details on search and document selection results - Presence of a flow diagram	31 (24.2) 4 (3.1)
Data extraction	Details on number of reviewers involved in extraction: - Extraction done by one reviewer - Extraction done by more than one reviewer (sharing, validation, or independent extraction)	55 (43) 18 (14.1) 37 (28.9)
Quality assessment	Quality assessment conducted - Critical appraisal tool specified	20 (15.6) 12 (9.4)
	Details on number of reviewers involved in assessment: - Assessment done by one reviewer - Assessment done by two reviewers	8 (6.3) 1 (0.8) 7 (5.5)
Synthesis	Narrative synthesis	128 (100)
	Meta-analysis	1 (0.8)
	Additional statistical analysis	2 (1.6)
	Assessment of evidence certainty	27 (21.1)
Revision and consultation	Internal revision (i.e., revision by reviewers from the organisation that produced the document)	85 (66.4)
	External revision (i.e., revision by reviewers outside of the organisation that produced the document)	35 (27.3)
	Consultations with experts, health professionals, managers, and representatives of professional orders	38 (29.7)

In general, most products reported information on the strategies used to search the literature. The large majority specified the databases used and more than half involved an information specialist to develop the search strategy. The main methodological modifications for the identification of the literature were to limit the number of databases and the searched language and period, Also, one characteristic of the reports analyzed is that almost all consulted the grey literature, which is a strategy typically omitted to expedite the process in rapid evidence synthesis. This can however be explained by the limited scientific data available at this point in the pandemic.

The reporting of RR was however insufficient for the other steps of the RR, with less than half that provided information on data selection, extraction, quality assessment, and synthesis. This lack of information prevents for clear conclusions to be drawn about the methodological adjustments used in the reviewed reports. The main modifications made at these steps, particularly early in the pandemic, were to have one reviewer for document selection and data extraction, and not assess the quality of the included studies and the certainty of evidence. Among the 20 reports that stated assessing the quality of studies, only 12 specified the critical appraisal tools used: AGREE II for guidelines, AMSTAR 2 or R-AMSTAR for systematic reviews, QUADAS 2 for diagnostic studies, AACODS for grey literature, and the Critical Appraisal Tool Kit that evaluates different types of quantitative studies. The synthesis was mainly narrative as found in the results, with some mentioning additional statistical analysis (*n* = 3).

Finally, more than half of the reports went through a review process, mainly internal revision. Also, nearly 30% provided information on consultations with experts, health professionals, managers, and representatives of professional orders, especially to discuss the recommendations put forward from the literature synthesis.

## Discussion

The COVID-19 pandemic has required evidence to be made available more quickly than usual to meet decision makers’ needs in a timely manner. This study documented 1) the challenges and adaptations made to produce RRs during the pandemic in Quebec, 2) the perceived usefulness, reported use and factors influencing the use of RRs and 3) the methodological adaptations made by organizations to produce RRs in Quebec.

The results highlight the HTA teams and organizations’ agility to deal with the many challenges encountered during the pandemic. For example, producers have increased their workloads, rapidly adopted new technological tools or work processes, collaborated more within and between organizations, developed scientific monitoring mechanisms, diversified the types of evidence used, adapted their methods to the requests' specificity, warned about the limits of RRs and updated their products.

When available at the right time, RRs have been useful in informing decisions in a context of uncertainty. RRs are described by evidence users as a reflective tool to support decision-making processes with scientific evidence or to justify decisions. However, their use is reported to be uneven across the system.

Among the analyzed documents produced between March 2020 and April 2021, insufficient information reported in the documents prevents us from drawing precise conclusions on the methodological adaptations used to speed up the evidence synthesis process. The main modifications reported were to limit the number of databases, to limit the searched language and period, to have only one reviewer for document selection and data extraction, and not to assess the quality of the studies and the evidence certainty. In this regard, teams and organizations expressed their discomfort during the pandemic to produce RRs since they streamlined their usual evidence synthesis methodologies, without clear guidelines at the outset on which choices to make to abbreviate the process.

### Collaboration efforts to avoid evidence synthesis duplication

Teamwork and collaboration and coordination between directorates or units within organizations and even between certain organizations were noted. Many reported the benefits of these intra- and inter-organizational collaborations facilitated by the pandemic emergency. However, according to participants, improvements can be made for a more optimal long-term coordination to optimize health system resources and avoid duplication in times of crises (e.g., centralization and requests sharing). In this regard, recent publications note that the lack of synergy at the local, regional, provincial, national and international levels during the pandemic may have led to evidence synthesis duplication efforts and, therefore, a waste of resources [38, 42–44]. It would be important to continue to understand the place of the Quebec health and social services system organizations in national and international networks, especially given the overlap observed between English and French evidence synthesis efforts [43].

### A highly political and mediatized context

The COVID-19 pandemic has put the spotlight on science, its usefulness but also its limitations and its role in government decision-making. In this study, evidence products and recommendations emanating from government institutes and agencies received much media attention. In Quebec, as elsewhere, the government’s scientific advisors and organizations acting in support of decision-making in the health and social services system received extraordinary visibility. This has brought to light for the public the inherent tensions between the political, economic and scientific dimensions of decision-making [39, 44]. Thus, the importance of continuously protecting the scientific integrity of organizations by keeping recommendations free from political pressure was noted by some participants. As recommended by Kuchenmüller et al. (2021, p. 2), these organizations must “*rely on systematic and transparent procedures to avoid conflicts of interest that may jeopardize their status as credible, independent sources of evidence, and expand regular foresight and rapid response activities in response to changing needs and contexts*” [44]. Strengthening independence and agility of these organizations, and improving communication and trust in government and institutions were some lessons learned, among others, in a recent study conducted in Quebec [39]. Organizations have experienced challenges navigating a highly mediatized environment where every production has the potential to be picked up by the media. A study recommends, among other things, that scientists and experts become more involved with journalists especially during a pandemic [45]. These authors note that given the proliferation of RRs, journalists may be less familiar with them. Because of the infodemia and misinformation that has circulated extensively regarding COVID-19, efforts to mobilize the best available evidence in an accessible manner remain necessary [44–46].

### System capacity for rapid evidence synthesis

The perceptions of the decision makers interviewed highlight the importance of supporting them in this emergency context. These results are consistent with other works that highlights the importance of ensuring that managers and policy-makers have access to the best available data, particularly in emergency situations [1, 2]. However, the pandemic has exacerbated this need to have access to evidence very quickly. As such, we need to determine when RR is appropriate. Certain criteria are proposed to guide this choice such as the topic of interest, stakeholders’ needs and expectations, decision-making urgency, and resources availability [14, 27, 34]. This also reinforces the relevance of strengthening the government’s institutional capacity in research, co-production, and evidence use and developing more sustained collaborative linkages between evidence producers and decision-makers [47, 48]. Recent years have shown us that the use of science is more important than ever to guide government actions, but also that its use in times of pandemic is even more difficult than it already is in more normal times because of many crisis governance challenges (e.g., uncertainty, high risk of loss, time constraints, conflicting values and principles) [49]. A recent commentary precisely discussed the thick walls between health research systems and decision-making spaces in Quebec and Canada and measures to better link evidence and decision-making in times of crisis [50].

Moreover, the gaps between RRs and the needs expressed by some decision-makers could also reflect a lack of stakeholders’ participation and deliberative processes to contextualize scientific evidence [51, 52]. These could not be carried out at the pandemic outset due to lack of time. Thus, reflections must continue to develop rapid participatory and deliberative mechanisms to integrate scientific evidence with experiential knowledge and contextual data during a pandemic emergency to develop optimal recommendations to support decision-making. Considering the unclear nature of data during a crisis like the COVID-19 pandemic, Smits et al., (2023) [50] argue the importance of integrating and considering both contextualized data and evidence from various disciplines (health disciplines and social sciences) and from international sources.

More broadly, the pandemic has highlighted the importance of having effective infrastructures in place and international collaboration networks to provide timely information to health systems. However, improvements could be made to the evidence synthesis mechanisms for managing priority demands and needs, coordination and monitoring. Organizations should continue to develop their capacity to anticipate and provide RRs to changing needs and contexts [44]. To this end, a system that keeps pace with scientific advances and constantly updates evidence syntheses and guidelines is essential for future health emergencies [53]. The development and effectiveness of living evidence synthesis methods, which have been increasingly used over the past five years, would also be an avenue for future studies in Quebec [48, 53, 54]. According to Tendal et al. [54], a living approach includes early prioritization of areas where guidelines are needed, ongoing monitoring of evidence, and frequent updating of recommendations, possibly on a weekly basis [54].

### Rapid evidence synthesis during non-pandemic context

The pandemic has increased the credibility and notoriety of certain organizations or HTA teams. This emergency context, and their great agility, increased awareness of their respective mandates and their added value. This notoriety may also come with higher expectations from decision makers since teams produced RRs in record time at the beginning of the pandemic. Further reflection is needed because this production rate is unsustainable with the current capacities of the teams. Collective reflections are needed on the use of RR products for non-pandemic decision processes and the efficient ways to produce them in a context of limited public resources. To this end, an increasing number of studies are focusing on technological developments based on artificial intelligence to optimize evidence synthesis processes. Some advances in this area before and during the pandemic are observed [42, 55]. Many initiatives experimented automation tools (e.g., COVID-NMA), crowdsourcing, shared platforms (e.g., LitCovid) or living strategies (e.g., eCOVID-19RecMap) [42]. These technological innovations will be important to consider in future research.

### Potential implications

Although this study was exploratory, certain implications for potential improvements are identified:

- Reinforce inter-organizational coordination in times of crisis, to ensure more optimal use of resources and expertise from HTA teams and organizations (e.g., mechanism for managing and keeping track of RR requests, a system-wide repository for reports, shared strategic and scientific evidence monitoring).
- Improve communication loops between teams producing RRs and requesters. It is important to take to time to clarify the request, so that responses can be better aligned with the needs of decision-makers. This would also enable a gradual transfer of knowledge in case the decision cannot wait.
- Develop guidelines or methodological benchmarks for rapid evidence synthesis in an emergency context, adapted to the realities of Quebec teams and organizations. Common standards and processes would help HTA professionals balance between speed and rigor.
- Make RR products more useful for decision-makers, given that decisions are particularly complex and multifactorial in times of crisis (e.g., guidelines for translating low-quality data into recommendations, mechanisms for involving more stakeholders such as patients and citizens, short but clear and operational documents).

### Study limitations

Some limitations of this study should be mentioned. First, the terms and the methodologies used to produce RRs varied considerably between teams and organizations, which could limit the specificity and generalization of the results. Also, the focus groups’ constitution in some settings (e.g., when the team manager was present) may have created a social desirability bias and influenced what the professionals felt they might or might not disclose. However, the researchers have made it clear that the project was not about evaluating their performance, but rather about lessons learned from their experience. Another limitation is the small number of participants recruited for the interviews. However, this allowed us to capture a diversity of perspectives and learn more about the potential of RRs in different decision-making contexts. Also, the snowball recruitment strategy used may have led to some selection bias. Those who agreed to participate are likely to have had a positive experience with RRs. Finally, the RRs analyzed are not necessarily representative of all the reports produced by the many organizations and teams involved in HTA in Quebec during the pandemic. Another limitation of the document analysis is that only 20% of the reports were verified by a second reviewer.

### Avenues for future research

Since organizations may have faced different challenges in subsequent waves of the pandemic, it would be important to further explore how they applied lessons learned from earlier waves. Other avenues worth exploring include: how to improve system capacity to deal with future crises by building on continuous evidence monitoring mechanisms and how to strengthen intra- and inter-organizational collaboration to avoid duplication of evidence synthesis efforts. It would also be important to assess whether the methodologies used to produce the RRs vary according to the types of decision-making needs expressed by requesters, and their intended use at which stage of the decision-making process (e.g., priority setting, policy formulation, implementation). This could help identify criteria for selecting appropriate product types based on context and question types. It would be useful to conduct a common reflexive approach to systematize the methodological benchmarks and to develop a typology of common evidence synthesis products. It would be important to conduct a study that explores in depth specific RR products, the challenges inherent to their realization context (e.g., nature of the issue, decision type, stakeholders involved) and their impact on decisions. It is also important to further our understanding of how RRs products can concretely inform practices, decisions, or policies during a pandemic and how, more generally, scientific evidence is integrated with other types of evidence and considerations for decision-making. The role of knowledge translation units in organizations should also be explored to highlight how they contribute or could optimize evidence use.

## Conclusions

This study highlighted the adaptive capacities of teams and organizations in Quebec to produce rapid evidence synthesis, and their role and usefulness in the decision-making process during the pandemic. When available at the right time, RRs were described as a valuable tool to support decision-making. The potential areas for improvement highlighted by this study include better coordination between organizations, improved communication loops between RRs producers and decision-makers, clarification of methodological benchmarks to balance speed and rigor, and a better understanding of how rapid evidence synthesis producers could better support decision-makers, given that decisions are particularly complex and multifactorial in times of crisis. A second research project is currently underway to develop a collective action plan to better prepare for a future health emergency in terms of system’s capacity to monitor and rapidly synthesize evidence.

## Data Availability

The datasets generated and analyzed during the current study are not publicly available due to integrity of participants but are available from the corresponding author on reasonable request.

## Abbreviations

HTA: Health technology assessment
IHSSC/IUHSSC: Integrated (University) Health and Social Services Centers
MHSS: Ministry of Health and Social Services
RR: Rapid response
